# Impact of floor covering on wheelchair rugby players: analysis of rolling performance

**DOI:** 10.3389/fspor.2023.1283035

**Published:** 2024-01-04

**Authors:** O. Vigié, A. Faupin, M-A. Ngo, C. Fauvet, D. Pradon

**Affiliations:** ^1^ Laboratory J-AP2S, UR201723207F, Toulon University, Toulon, Var, France; ^2^ Gredeg—Campus Azur du CNRS250 F06905 Sophia Antipolis Cedex, Nice, Alpes Maritime, France; ^3^ Pole Parasport—ISPC Synergies, CHU Raymond Poincaré, APHP, Garches, Iles de France, France; ^4^ EndiCap UMR 1179 INSERM–Paris-Saclay University, Ile de France, France

**Keywords:** sports equipment, field testing, disabled athletes, wheelchair rugby players, floor surface

## Abstract

**Introduction:**

Despite the increased interest in indoor wheelchair sports in many countries, research on the effect of floor coverings on sports performance is limited. Currently, there are no specific guidelines for covering characteristics for wheelchair sports, whether for competitive or recreational purposes. This study aimed to determine the impact of floor coverings on the biomechanical parameters of manual wheelchair propulsion for wheelchair rugby practice.

**Methods:**

Ten wheelchair rugby players performed 6 maximum-velocity sprints over 20 meters, with a 20-second recovery time between sprints, on 3 different coverings, using their personal sports wheelchairs. The coverings were: wood parquet, Gerflor TX System Endurance®, and a plastic synthetic covering (balatum). Performance and propulsion technique variables were collected using inertial measurement units (265 Hz, Kinvent, France). Additionally, rolling resistance quantification tests were conducted on each covering.

**Results:**

Rolling resistance was lowest on the wood parquet, with an average value of 3.98 ± 0.97 N. Best sprint performance was achieved on the wood parquet. The fatigue index on the parquet was significantly lower than on the balatum (*p* < 0.05).

**Discussion:**

Our results highlight that floor surface influences both performance and propulsion technique variables. Therefore, we recommend performing wheelchair rugby training on wood parquet to optimize performance. It is also important to consider the impact of different coverings on sprint performance when organizing player rotations to maintain a high level of competition during tournaments.

## Introduction

Wheelchair sports are influenced by the surface on which the sport is practiced. Different floor coverings create different amounts of rolling resistance. For example, rolling resistance is higher on carpet than on linoleum or wood parquet (1). The International Wheelchair Rugby Federation recommends the use of hard wood coverings (IWRF European Championship Division A, 2022 IWRF Asia-Oceania Championship). These recommendations are based on the requirements set by the International Wheelchair Basketball Federation (IWBF), which implicitly follow the regulations established by the International Basketball Federation. As a result, wheelchair rugby players practice on surfaces initially intended for able-bodied athletes despite the obvious functional differences and specific needs of wheelchair rugby players. Wheelchair rugby requires a high capacity to rapidly accelerate (2, 3). This is essential to achieve fast sprint performances. This capacity can be measured using various performance parameters such as maximum velocities and accelerations (4, 5), cycle time and cycle frequency (6). Understanding and analyzing these biomechanical parameters is useful for the development of training strategies to improve the efficiency and performance of wheelchair rugby players. Another consideration is injury; wheelchair rugby players are at a high risk of developing musculoskeletal disorders because of excessive strain on their upper limbs during practice, as well as rolling resistances generated during movements and maneuvers (7). Fatigue during propulsion can lead to the use of compensatory strategies, which further increases the risk of shoulder injuries (8).

Although biomechanical and physiological analysis of wheelchair rugby has shed light on the impact of players' physical and technical abilities or disabilities (9) and the type of wheelchair on performance (10), no studies have focused on the effect of the floor covering on sports performance. Therefore, research on the effects of floor covering on wheelchair propulsion is needed to develop recommendations that address the needs of wheelchair rugby players based on relevant scientific data. This study aimed to quantify the effect of the floor covering on the biomechanical parameters of sprinting. We anticipate that the results will serve as a catalyst for parasport federations to deliberate upon the playing surface factor of performance. The specific objective of this study was to quantify and compare the impact of wood parquet, a linoleum-type covering (balatum) and a Gerflor TX System Endurance covering on biomechanical variables, performances variables and fatigue index during wheelchair rugby propulsion. We hypothesized that the wood parquet surface would be the most suitable for wheelchair rugby practice, ie, that rolling resistance and fatigue index would be lowest, performance time shortest and biomechanical variables would be more optimal than on the other 2 coverings.

## Materials and methods

### Participants

Ten members of the Rugby Fauteuil Club Toulon Provence Méditerranée (Toulon Provence Méditerranée Rugby Club, France) participated in the study, including 8 men and 2 women with the following conditions: cerebral palsy (*n* = 2), paraplegia (*n* = 2), tetraplegia (*n* = 1), spina bifida (*n* = 1), limb deficiency (*n* = 1) and able-bodied (*n* = 3). Some of the participants played wheelchair rugby (originally murderball, known as quad rugby in the United States) and the others played wheelchair rugby league (a wheelchair-based version of rugby league).

The players had between 1 and 10 years of experience in competitive practice at a national level. They competed at the national level 3, trained twice a week, and participated in a total of 15 annual matches. (Table 1) The experimental protocol was approved by the Institutional Research Ethics Committee in Physical and Sports Activities Sciences and Techniques (approval number: IRB00012476-2020-08-04-55). Prior to participating, all participants signed an informed consent form.

**Table 1:** Anthropometric characteristics and etiology of disability of participants.

Participants	Sex (M/F)	Age (years)	Mass (kg)	Disability
WR1	M	25	68	NONE
WR2	M	45	85	SCI
WR3	M	39	70	Cerebral palsy
WR4	M	23	75	NONE
WR5	M	21	78	Spina bifida
WR6	M	44	125	SCI
WR7	F	21	59	NONE
WR8	M	13	36	Cerebral palsy
WR9	F	29	45	SCI
WR10	M	49	75	Limb defiency

M, male; F, female; SCI, Spinal Cord Injury.

### Surface characteristics

During the protocol, we ensured the integrity and cleanliness of the surfaces. We did not conduct any testing of their mechanical properties, instead, we relied on the information provided in the surface's reference documentation. The surface types were:

- a synthetic surface resembling a soft floor similar to balatum (which is similar to linoleum) (BAL), of which the specific characteristics were unknown,
- a wood parquet floor (WP) that meets the NF EN 14904 (NF P 90-203) standards required by the French Basketball Federation for the practice of this discipline,
- a “Gerflor TX System Endurance” covering (GER-TX) placed over the wood parquet floor.

The exact age of the surfaces remains uncertain; however, as per the sports complex management, the WP and the BAL were estimated to be under 5 years old, while the GER-TX surface was estimated to be under 1 year old.

### Quantification of rolling resistance: deceleration test

We conducted a deceleration test inspired from Bascou et al. (11) to quantify rolling resistance on each surface. This test involves quantifying wheelchair deceleration when no propulsion force is applied. The test is organized in 5 steps: (1) a static phase of 2 s on a fixed starting mark on the floor, (2) a manual push by a third person to set the manual wheelchair in motion between 2 marks placed 5 meters apart, (3) a deceleration phase while ensuring the proper trajectory of the manual wheelchair, (4) a manual stop at 5 meters, (5) a static phase of 2 s. To ensure an equal distribution of masses on the seat, several tests were conducted with a 30 kg weight placed either at the front or at the back of the seat of each wheelchair.

For each condition, 6 deceleration test trials were conducted. Each player used their own sports wheelchair which was not modified for this test. The wheel camber angle was set at 18 degrees, but the wheel sizes varied between 24 and 25 inches, and the tire pressure ranged from 7.5 to 8 bars (measured using a manometer). The wheelchairs were fitted with the athletes' tires, and these remained consistent throughout the protocol. The tires used for the deceleration test were identical under all conditions. Although the tires were not brand new, they had been recently installed, and their integrity and condition were verified before the testing protocol. We made the decision to rely on the manufacturer's recommendations for tire pressure to ensure the proper execution of our testing protocol.

### Athletic performance: repeated sprint test

The repeated sprint test involves performing multiple, maximum intensity sprints with recovery periods between sprints (12). Using their own sports wheelchair, the athletes performed 6 maximum velocity sprints over 20 meters, with a 20-second recovery time between sprints. The sprints started from a stationary position, following a warm-up that adhered to the usual training guidelines, including at least 3 progressive maximal accelerations over 20 meters. The order of the sprints on each floor covering was randomized. Between each session of repeated sprints, the participants had a 10-minute rest (13). All tests were performed on the same day.

Each wheelchair was equipped with 3 inertial measurement units: 1 on each wheel and 1 on the frame of the wheelchair (14). The units consisted of 3 types of sensors that acquired data at 265 Hz: 3 accelerometers to measure movement acceleration (in m/s²), 3 gyroscopes to measure angular velocity (in rad/s), and 3 magnetometers to indicate orientation relative to the Earth's magnetic field (Kinvent, France). A specific script was developed for the processing and analysis of the signals using Matlab software (MathWorks, MA, USA).

### Data analysis

We determined deceleration values by deriving the linear velocity of the wheels. Subsequently, rolling resistance was computed using an equation using: the total deceleration force, which consists of rolling resistance force, air resistance force, gravitational force, and inertial friction force. The key variables include: *m* for the combined mass of the wheelchair and the additional 30 kg masses, a for the deceleration value, and *v* for linear velocity (15).

The linear displacement velocities for each rear wheel were calculated from the angular velocity recorded by the non-slip inertial measurement unit and the wheel radius (16). The data collected by the IMUs were processed using a specific script involving calculation of the average of all variables for the left and right wheels for each push. We applied a fourth-order zero-lag Butterworth filter to eliminate random noise, followed by a low-pass filter with a cutoff frequency of 8 Hz and order 4 to process the signals (17).

**Figure 1:**
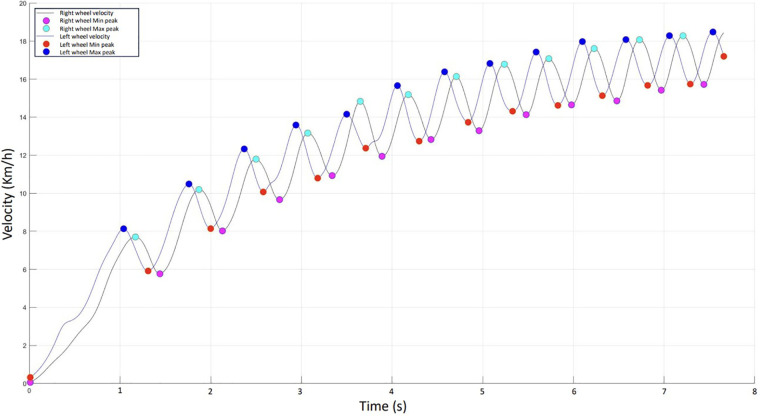
Example of a sprint recording: velocity curves for the right and left wheels as a function of time in seconds.

Stable velocity was calculated from the last 5 propulsions. Acceleration at the start of the sprint was calculated from the first 3 propulsions. Propulsion cadence was defined as the number of propulsion cycles performed per minute. The propulsion phase time was defined as the time between the first minimum peak and the maximum peak, and the cycle time was the time between 2 minimum peaks (Figure 1). Sprint times were measured using the WITTY GATE photoelectric cell system (Rizzetto, Guide Version 1.5).

Additionally, a fatigue index was calculated across the 6 sprints for each player using the following equation validated by scientific literature that incorporates the best sprint performance time and the last sprint performance time (18): FI = ((|Best − Last|)/Best) × 100 (%).

Statistical analysis was conducted using JASP 0.16.0.0. The Shapiro-Wilk test was used to assess whether the variables followed a normal distribution, and the Bartlett test was used to evaluate the homogeneity of variances. Among the analyzed variables, only 2 followed a normal distribution. Therefore, a Friedman test (non-parametric) was conducted. A *post hoc* Friedman-Nemenyl test with Bonferroni adjustment was applied when significant differences were found. Kendall's W was calculated to indicate effect sizes. For the rolling resistance test, after verifying the normal distribution of the data and the homogeneity of variances, we conducted a one-factor repeated measures ANOVA. The significance level was set at *p* < 0.05.

## Results

### Rolling resistance test

**Figure 2:**
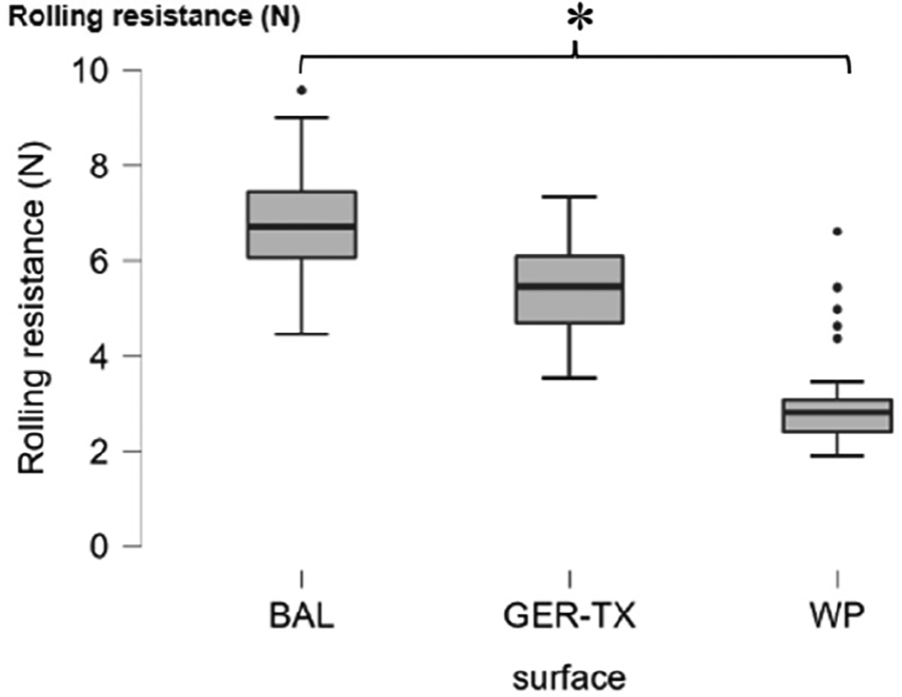
Wp, Wood parquet flooring; BAL, soft floor covering similar to linoleum; GER-TX, Gerflor TX system endurance ®; *, significant difference found with *post hoc* pair wise comparisons with Bonferroni adjustment, *p* = 0.05.

The average rolling resistance was 60% higher on the BAL and 106% higher on the GER-TX surface than on the WP (Figure 2).

### Analysis of performance according to floor covering

The results of the statistical analysis are presented in Table 2. The best sprint performance was during the first sprint in 70% of cases. Sprint time differed significantly between WP, BAL (*p* < 0.001) and GER-TX (*p* < 0.05). Maximum velocity was significantly higher on WP than on GER-TX and BAL (*p* < 0.05). The number of propulsion cycles varied depending on the covering, with a significant difference between WP and GER-TX (*p* < 0.05).

**Table 2:** Result of the kinematic variables for the best sprint.

	Descriptive data (*median (Q1; Q3*)			Surface effect			Post hoc Friedman Test		
Variables	BAL	GER-TX	WP	*χ* ^2^	*p* value	*W*	*BAL _WP*	*GER-TX _WP*	*GER-TX_ BAL*
Peak Velocity [m/s^1^]	3.84 (3.45; 4.44)	3.99 (3.47; 4.32)	4.22 (3.71; 4.61)	*12*.*2*	***<0*** **.** ***01***	0.61	*0*.05*	*0*.*108*	*1*
Vstab [m/s^1^]	3.14 (3.42; 4.31)	3.93 (3.45; 4.16)	4.14 (3.62; 4.39)	*13*.*4*	***0*** **.** ***001***	0.67	*<0*.01**	*<0*.05*	*0.835*
AccMeanStart [m/s^2^]	1.39 (1.15; 1.68)	1.44 (1.20; 1.61)	1.61 (1.33; 175)	*8*.*97*	***<0*** **.** ***05***	0.45	*0*.033*	*0*.*108*	*1*
Time [s]	7.11 (6.40; 7.77)	6.82 (6.42; 7.70)	6.62 (6.01; 7.27)	*15*.*2*	***<0*** **.** ***001***	0.76	*<0*.001**	*<0*.05*	*1*
Cycle frequency [cycle.s^−1^]	1.93 (1.79; 1.97)	1.83 (1.65; 1.96)	1.87 (1.81; 1.92)	*7*.*8*	***<0*** **.** ***05***	0.39	*0*.*178*	*<0*.05*	*1*
Push time (PT) [s]	0.29 (0.27; 0.31)	0.30 (0.27; 0.31)	0.28 (0.26; 0.30)	*11*.*4*	***<0*** **.** ***005***	0.57	*0*.*22*	*0*.01**	*0.439*
Cycle time (CT) [s]	0.47 (046; 0.48)	0.49 (0.47; 0.52)	0.46 (0.42; 0.50)	*6*.*37*	***<0*** **.** ***05***	0.32	*0*.*66*	*0*.*66*	*0.06*

WP, Wood parquet flooring; BAL, soft floor covering similar to linoleum; GER-TX, Gerflor TX System Endurance ®; M, median; Q1, first quartile; Q3, third quartile; *χ*^2^ is the test statistic and Kendall's W is the effect size for the non-parametric Friedman test. *p*-value was fixed at 0.05.

Significant differences between WP, BAL and GER-TX are showen by *p < 0.05, **p < 0.01.

**Figure 3:**
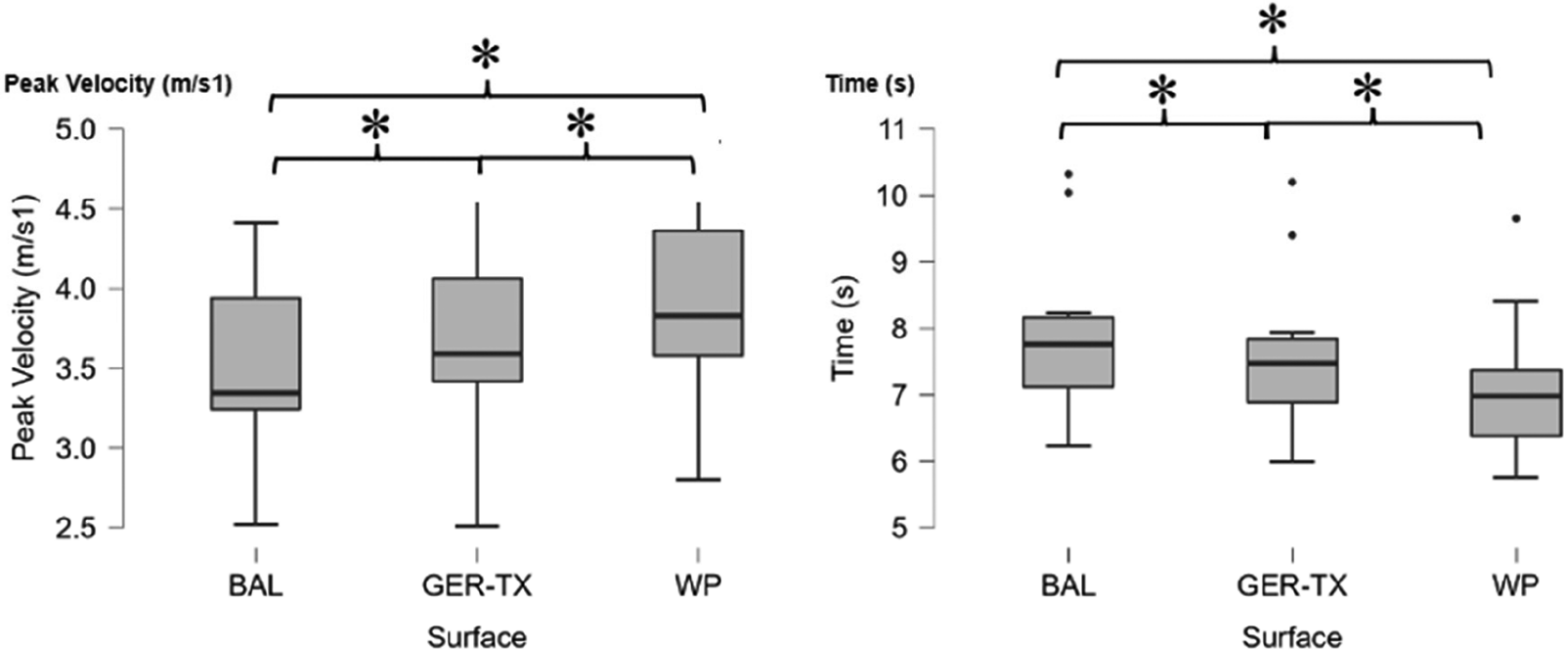
Result of the peak velocity and time for the sixth sprint. WP, Wood parquet flooring; BAL, soft floor covering similar to linoleum; GER-TX, Gerflor TX System Endurance ®; *, significant difference found with *post hoc* pair wise comparisons with Bonferroni adjustment.

Performance decreased with the repetitions of the sprint test. Sprint times were longer for the last than the best sprint. During the last sprint, the maximum velocity reached was significantly higher on the WP. Maximal velocity during the best performance sprint was also highest on the WP (Figure 3).

The fatigue index was significantly higher for BAL than WP (*p* = 0.048). Specifically, it was highest for BAL: 9.80% (±6.71) then GER-TX: 7.50% (±5.30), and lowest for WP: 5.90% (±4.18).

## Discussion

This study investigated the influence of floor coverings on wheelchair rugby player performance. As we hypothesized, the results revealed that rolling resistance, sprint times, and fatigue index were reduced on wood parquet flooring compared to linoleum or synthetic plastic coverings.

### Effect of a repeated sprint field test on wheelchair rugby players

Wheelchair rugby players can play up to 32 min on the court, and maintaining their performance throughout the match is crucial for the outcome. We decided to evaluate the impact of floor covering using a repeated sprint field test because this test is similar to conditions encountered in elite-level wheelchair rugby matches (19).

As expected the repeated sprint field test impacted on the wheelchair rugby players: our findings revealed a decline in performance variables as the test advanced, irrespective of the floor covering. Sprint time increased throughout the tests and in the final trial, the sprint time was significantly longer than in the first trial for all 3 coverings. This loss of performance over sprint repetitions is corroborated by the study conducted by Bakatchina et al. in (15), using a similar repeated sprint protocol.

Nonetheless, the fundamental interest of this study lies in elucidating the impact of surface coating on the movement of wheelchair rugby practitioners. By undertaking a comparative analysis of outcomes achieved on distinct surfaces, we are capable of delineating the surface texture that induces the least disruption, given the imperative of upholding performance throughout the entirety of the game.

### Impact of surface on rolling resistance

Wheelchair movement on any surface is subject to rolling resistance. Hofstad et al. (20) determined 3 types of rolling resistance: aerodynamic resistance, internal rolling resistance generated by different elements of the wheelchair, and external rolling resistance generated by the characteristics of the wheelchair's contact with the ground. According to Chua et al. (1), rolling resistance on a carpeted floor is significantly higher than on linoleum or wooden parquet flooring. Identifying and understanding of these different types of rolling resistance is crucial for optimizing manual wheelchair propulsion efficiency. Our results indicate that the WP flooring has the lowest rolling resistance. Given the negative correlation between wheelchair rolling resistance and speed during sprints (21), the speed of wheelchair athletes should be higher on WP than on surfaces with higher rolling resistances.

### Impact of floor covering on wheelchair rugby propulsion efficiency

#### Performance variables

The different increases in fatigue index with repeated sprints for each floor covering found in our study further underline the importance of the flooring on performance. It is essential that studies of wheelchair athlete performance specify the type of floor covering on which the experiments were conducted, however this is seldom the case. For example, Gee et al. (19) reported timed 20-meter sprint performances of 7.19 ± 0.89 s in wheelchair rugby players.

This value is lower than that found on the WP but similar to the BAL in our study. However, because the surface-type was not indicated, it is not possible to compare our results with theirs. More recently, the study conducted by Bakatchina et al. (10) allows us to more precisely position the performance of our athletes relative to other studies, based on their playing position. The authors reported that the median (Q1; Q3) timed performance for a 20-meter sprint on a wooden parquet floor is 7.42 s (7.12; 8.26) for attack wheelchair users and 7.31 s (6.57; 7.72) for defense wheelchair users.

It is important to note that the maximum velocity achieved by an athlete is an indicator of their ability to generate high velocities over a short distance. This parameter is widely measured and interpreted by sports coaches due to its importance for performance evaluation. In our study, the highest performance was achieved on the WP with a median maximum velocity of 4.22 m/s (3.71; 4.61). During a quarter of play in international wheelchair rugby competitions, players classified by the International Wheelchair Rugby Federation as 3.0-point or 3.5-point reached a mean maximum velocity of 3.82 ± 0.31 m/s (22). Although the majority of international wheelchair rugby competitions take place on wood parquet flooring, the lack of specification of the nature of the floor covering in that study makes direct comparison of the maximum velocities obtained difficult. Indeed, differences in environmental conditions and test protocols could also explain the discrepancy between our results and the maximum velocities observed during matches. In addition to the impact of the floor covering, factors such as temperature and humidity can also affect player performance during a match (23).

In sports played on a small court, such as wheelchair rugby, it is important to analyze the efficiency of initial pushes and movements. Wheelchair rugby players exert intermittent efforts during matches and must repeatedly exert maximal efforts (24, 25). These intermittent efforts lead to a temporary state of fatigue, resulting in a decrease in performance (8, 26). In the last sprint, acceleration at the start was higher on the WP than on the other floor coverings, although the difference was not statistically significant. The capacity to initiate a sprint from a stationary position is considered a critical aspect of performance in indoor manual wheelchair sports (27) because of its relevance to winning ball conflicts during the game in which match performance translates to the ability to outmaneuver opponents. In our study, the difference in performance on the different surfaces, even when the subject was potentially fatigued, validates our hypothesis that the floor covering impacts on the ability to sustain effort.

#### Biomechanical variables

Wheelchair propulsion cycle time, push time, cycle frequency and cadence provide indications of propulsion efficiency as well as the risk of musculoskeletal injury (28, 29). Propulsion technique variables are also crucial when analyzing performance maintenance and compensation for fatigue (8). Our results showed that on the WP, the time required to complete a full propulsion cycle was shorter than on the other floor coverings. This indicates that on the WP, wheelchair rugby players performed pushrim movements more rapidly than on other floor coverings.

Furthermore, the duration of each propulsion cycle decreased proportionally with the increase in velocity on each floor covering.

These results are consistent with previous reports of wheelchair propulsion on a treadmill: a decrease in cycle time and push time is expected with an increase in movement speed (30). In another study, the reduction in cycle time was explained by a reduction in push time while maintaining a constant push angle (31).

The surface-type may also affect the propulsion strategy. The propulsion cadence, expressed as the number of cycles per minute, was significantly higher on the WP than the GER-TX. Since the WP was associated with less rolling resistance, we suggest that the propulsion technique adopted by the participants was higher cadence rather than maximal power. Our results are consistent with those of previous studies that found a positive correlation between propulsion frequency and velocity (30). Propulsion cadence is inconsistently adjusted by wheelchair athletes when rolling resistance increases (32). Optimal performance of para-athletes may be linked to their ability to generate significant power with their upper limbs while reducing the frequency of their propulsion movements (33). Those findings, along with our results, could be particularly relevant for wheelchair rugby players engaged in competitive sports. Indeed, since the propulsion cadence was higher on the WP, enhancing power development capacity and reducing propulsion cadence may improve performance on that flooring.

We have identified a significant difference in the number of propulsion cycles executed on the WP and GER-TX surfaces. It is worth highlighting that there is no significant difference in the time duration of each cycle. This observation implies that players made modifications to their propulsion parameters to sustain their performance levels. Consequently, they were compelled to execute a greater number of cycles owing to the prolonged duration of the sprints. This observation holds considerable significance within the context of performance analysis, as it signifies that athletes adapted their propulsion strategies to mitigate the impact of surface disparities, which can exert a substantial influence on competitive results.

Players may adapt their propulsion technique to maintain their speed. Studies have shown that the same maximal speed can be achieved using different propulsion techniques. The technique parameters that are varied include propulsion cadence, force applied to the handrim, and different propulsion recovery times (34). This knowledge challenges the notion of performance degradation, since the variance in propulsion technique parameters does not necessarily imply lower performance levels. Nonetheless, specific criteria have been identified in the scientific literature, enabling researchers to offer recommendations concerning the optimal propulsion technique to be adopted depending on the specific sports discipline (35, 36, 37).

#### Fatigue index

The floor covering exerted a significant influence on the fatigue index. As a reminder, the fatigue index was computed according to the player's timed performance. Our findings indicate that the fatigue index value was significantly higher on the BAL than on the WP. This implies a notable decline in performance.

Once again, our results underscore the importance of considering the floor covering in the analysis and formulation of gameplay strategies during wheelchair rugby practice. These results further substantiate our hypothesis of superior performance quality and enhanced gameplay comfort for wheelchair rugby players on WP.

Moreover, given that wheelchair rugby players have disabilities, it is imperative to focus on their functional capabilities. Indeed, functional limitations, such as muscle weakness or reduced joint mobility, can significantly affect their ability to maintain an optimal propulsion technique. The scientific literature consistently shows performance disparities, propulsion asymmetries, and divergent propulsion techniques among players of different categories (15).

Consistent with prior scientific observations, our results underscore the necessity of providing particular attention to players classified as “low point” because of their more limited functional capacities. It is highly probable that these individuals will experience a more pronounced deterioration in performance over time. Our findings provide evidence that the playing surface impacts on the decline in performance, and we put forth the hypothesis of a potential interplay between the “surface” factor and the “functional capacities” factor. However, due to the limited size of our sample, definitive conclusions cannot be drawn.

### Implications

In the planning of a wheelchair rugby event, it is crucial for the coaching staff to be fully aware of the impact of the floor covering when making decisions about player substitutions during the competition. The possibilities for substitutions within a team and the tactical aspects related to the coach's selection of the 4 players on the court are numerous. Players classified as 0.5 point are the most common on the field during matches (38). Performance degradation over time is known to be significantly greater in players with lower functional capacity, i.e., with lower classifications (10, 19). Based on our findings, we recommend that coaches increase the rest periods of players, depending on the type of surface. This recommendation aims to optimize performance while preserving the integrity of the musculoskeletal system and preventing the onset of pain or discomfort.

### Limitations

This study has several limitations. Mainly, we did not evaluate tire traction on the playing surface or player behavior during turns. These factors should be prioritized in future research efforts, as they have the capacity to augment our understanding of the physical and technical demands inherent in wheelchair rugby when played on diverse surfaces. Furthermore, the lack of technical information available about the synthetic surface resembling a soft floor similar to balatum somewhat limits understanding of the impact of this floor covering on performance.

## Conclusion

To our knowledge, this is the first study to provide information about the effect of the surface on wheelchair propulsion mechanics and sprint performance.

Our results lead us to recommend that wheelchair rugby should be played on wood parquet flooring, rather than a linoleum-type or GER-TX covering. Performance is more reproducible on wood parquet, and the temporal variables suggest that the risk of musculoskeletal injury may be lower. However, if that is not possible, we advise coaches to increase player rotations among those with lower functional classifications to reduce the fatigue induced by the flooring.

We sincerely hope that this study will encourage parasport federations to consider the surface of play and that it will raise awareness among sports infrastructure manufacturers and decision-makers about the importance of providing access to sports in conditions equivalent to those of able-bodied athletes.

## Data Availability

The original contributions presented in the study are included in the article/Supplementary Material, further inquiries can be directed to the corresponding author.
